# Supplementary figures and images for: Plexins promote Hedgehog signaling through their cytoplasmic GAP activity
Source: eLife. 2022 Sep 28;11:e74750. doi: 10.7554/eLife.74750 (PMC9553217; doi:10.7554/eLife.74750)

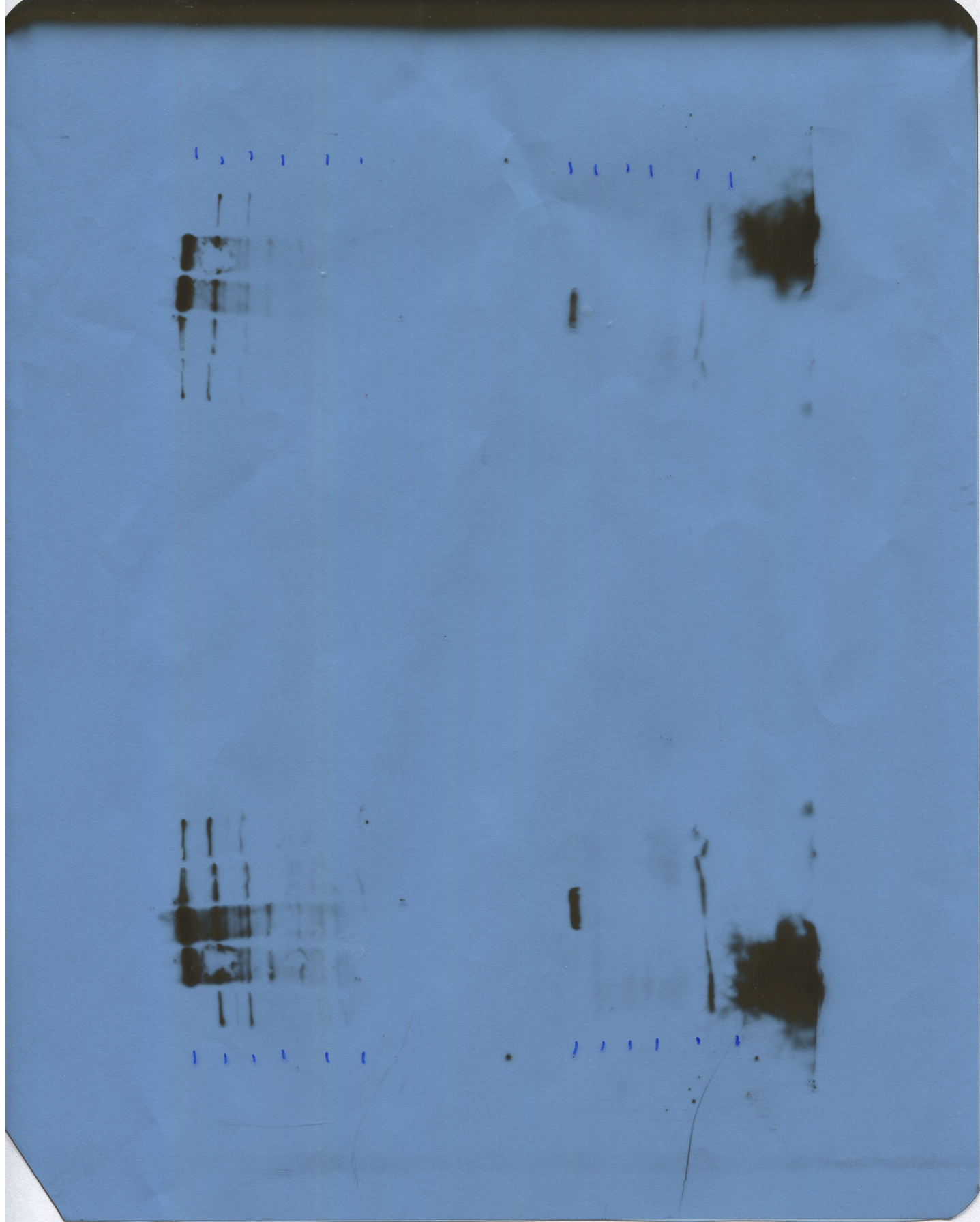

Supplement: Figure 2—source data 2. [file elife-74750-fig2-data2.pdf]

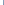

9

u!u!

[illegible]

1 1 1 d 1 1  
6

Supplement: Figure 2—source data 3. [file elife-74750-fig2-data3.pdf]

PDS, PIXNA1, PIXNAITMCD, PIXNAIACD  
 Lanes 1-4 : ~~by~~ supernatants  
 5-8 : Lysates (3T3)  
 MS1961 &  $\beta$ -tub

5 sec ~~10 sec~~  
 250 -  
 150 -  
 100 -  
 75 -  
 50 -  
 40 -  
 30 -  
 20 -

1 1 1 1 1  
 1 1 1 1 1

9/21/17

325C 5.81M17

NIMS

5.81M17

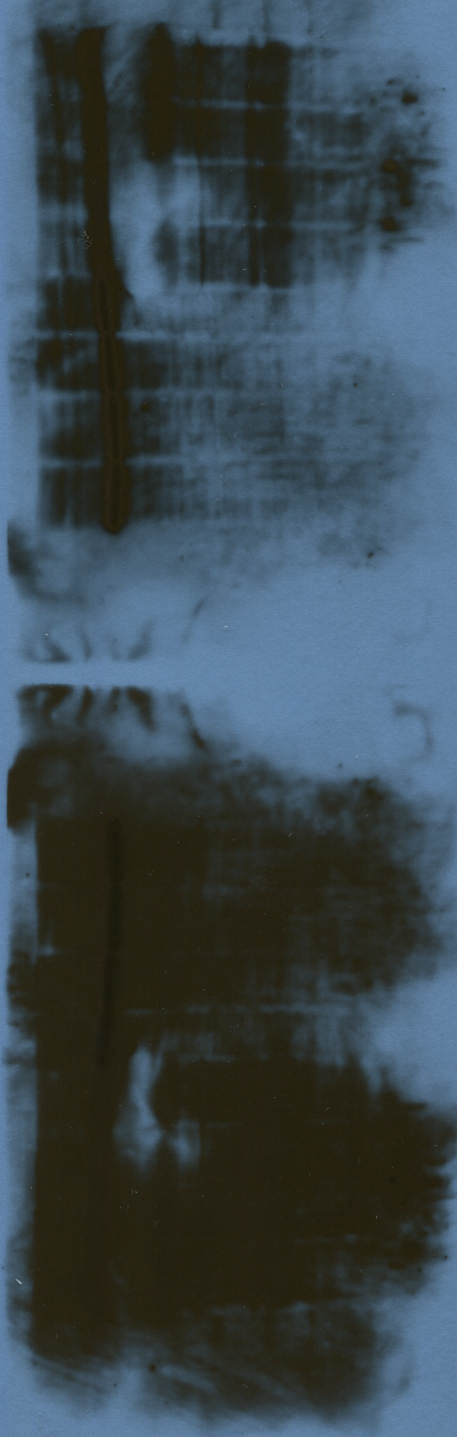

Supplement: Figure 2—source data 4. [file elife-74750-fig2-data4.pdf]

pCIG  
MYC::PLXNA1  
MYC::PLXNA1 $\Delta$ TMCD  
MYC::PLXNA1 $\Delta$ CD

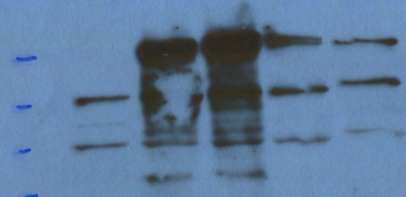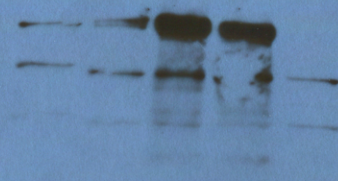

Supplement: Figure 2—source data 5. [file elife-74750-fig2-data5.pdf]
