# Supplementary material for: Plexins promote Hedgehog signaling through their cytoplasmic GAP activity
Source: eLife. 2022 Sep 28;11:e74750. doi: 10.7554/eLife.74750 (PMC9553217; doi:10.7554/eLife.74750)
Supplement: Figure 2—source data 6. [file elife-74750-fig2-data6.pdf]

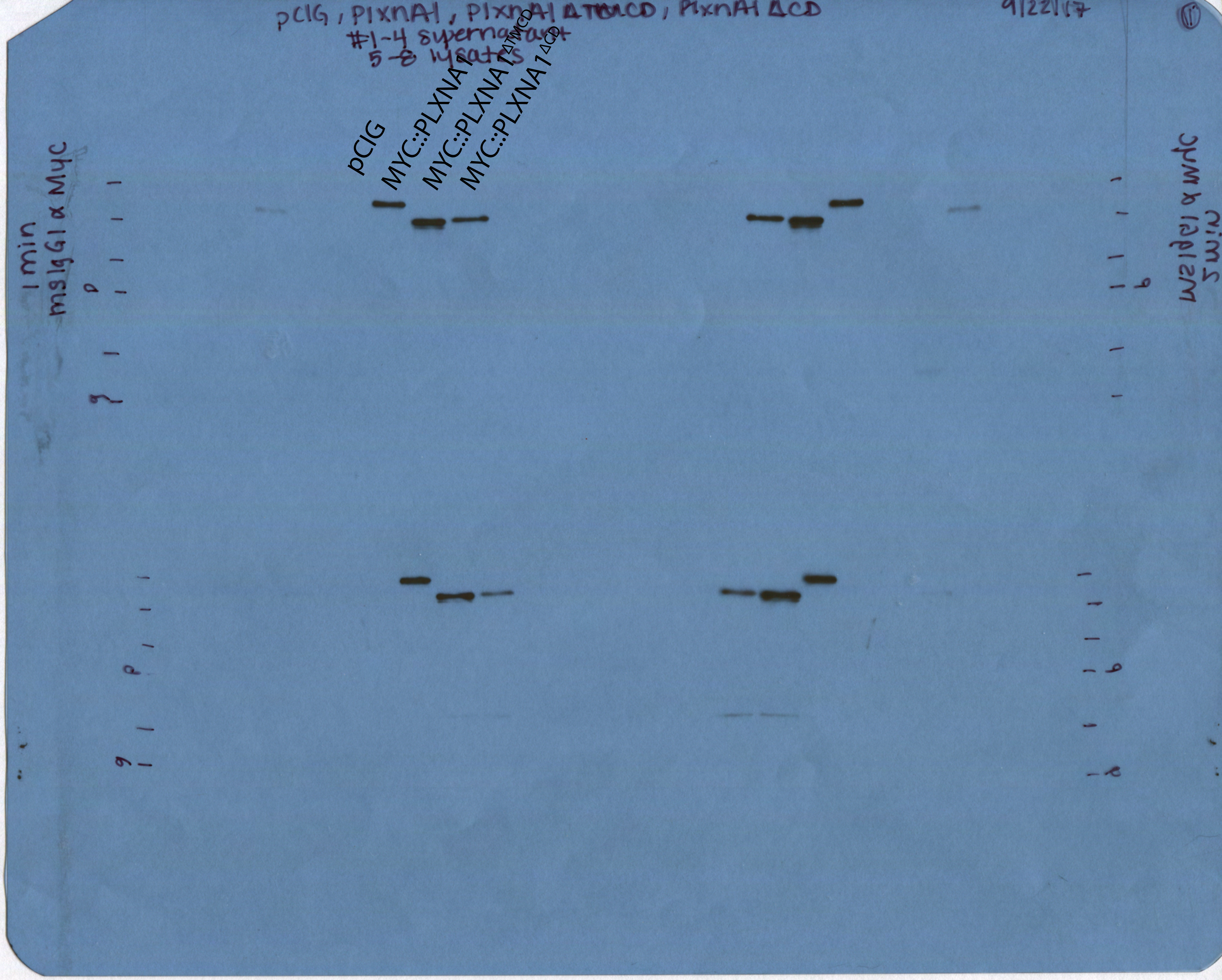
pCIG, PlxnA1, PlxnA1 ΔTMCD, PlxnA1 ΔCD
9/22/17
#1-4 supernatant
5-8 lysates
pCIG
MYC::PLXNA1
MYC::PLXNA1ΔTMCD
MYC::PLXNA1ΔCD
1 min
msIgG1 α Myc
