# Supplementary material for: Plexins promote Hedgehog signaling through their cytoplasmic GAP activity
Source: eLife. 2022 Sep 28;11:e74750. doi: 10.7554/eLife.74750 (PMC9553217; doi:10.7554/eLife.74750)
Supplement: Figure 2—source data 7. [file elife-74750-fig2-data7.pdf]

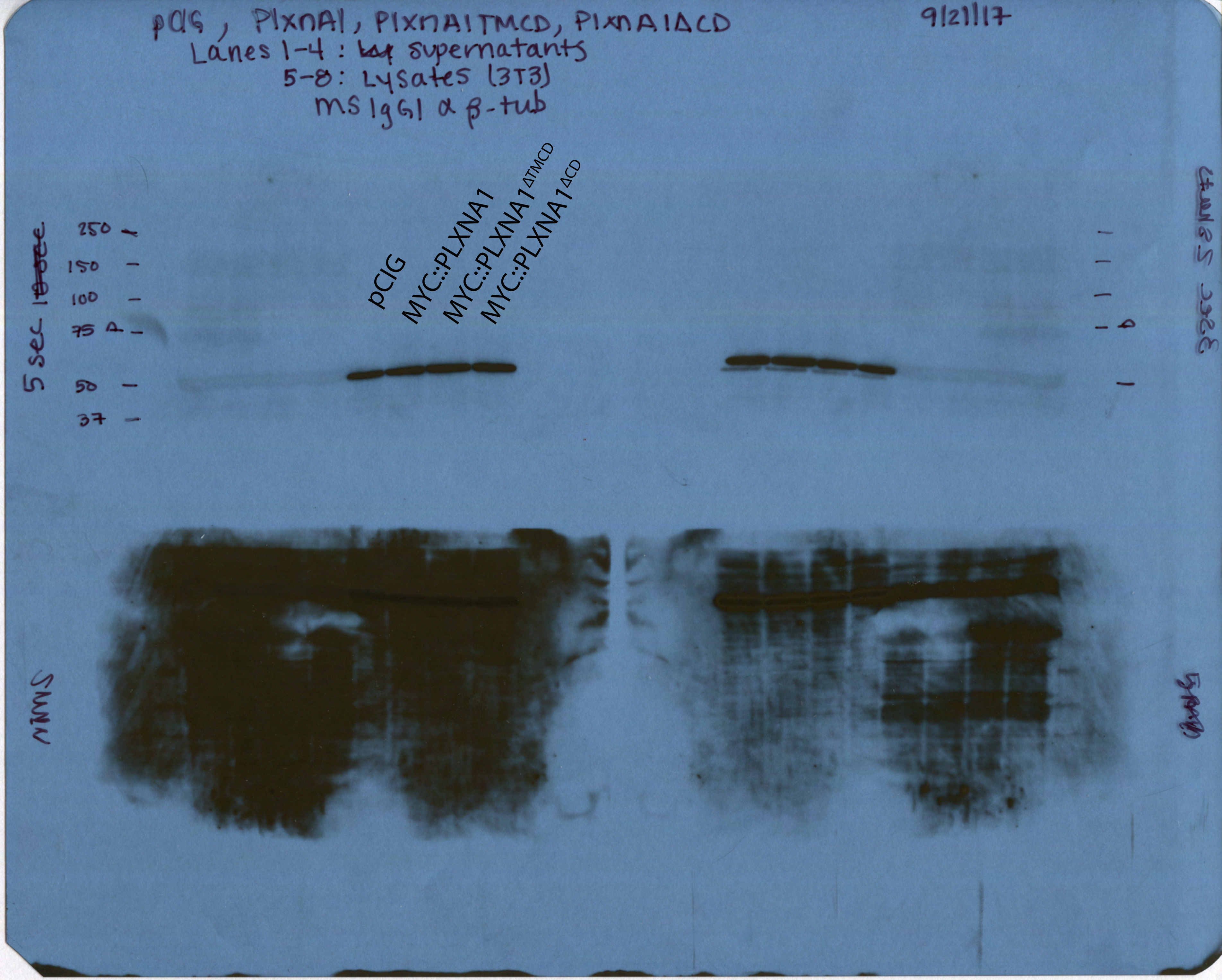
pCIG, PlxnA1, PlxnA1TMCD, PlxnA1ΔCD
9/21/17
Lanes 1-4 : supernatants
5-8: Lysates (3T3)
MS IgG1 α β-tub
5 sec
250
150
100
75
50
37
pCIG
MYC::PLXNA1
MYC::PLXNA1ΔTMCD
MYC::PLXNA1ΔCD
